# Clinical efficacy evaluation of TiRobot-assisted minimally invasive treatment for calcaneal fractures based on enhanced recovery after surgery principles

**DOI:** 10.3389/fsurg.2025.1673655

**Published:** 2025-10-02

**Authors:** Dai Yonghong, Hong Shi, Wang Shiheng, Zeng Yanhui, Yang Kuangyang, Wang Shaoyun

**Affiliations:** 1 The Eighth Clinical Medical College of Guangzhou University of Chinese Medicine, Foshan, Guangdong, China; 2 Foshan Hospital of Traditional Chinese Medicine, Foshan, Guangdong, China

**Keywords:** navigation, minimally invasive, calcaneal fracture, closed reduction, robotic surgery, rapid postoperative rehabilitation

## Abstract

**Background:**

Calcaneal fractures were the most common hindfoot fractures, with most being intra-articular. Compared to fractures in other locations, calcaneal fractures had poorer prognoses. These fractures significantly reduced patients' quality of life, causing long-term chronic pain and functional impairment. Rapid recovery was crucial for restoring patients' normal life and work abilities. Advances in robotic technology provided a new surgical approach to promote faster rehabilitation for calcaneal fractures.

**Methods:**

A retrospective analysis was conducted on the medical records of 20 patients with calcaneal fractures who underwent surgery at Foshan Hospital of Traditional Chinese Medicine from October 2020 to November 2024. Based on surgical methods, patients were divided into the robot-assisted group (10 cases) and the control group (10 cases). The robot-assisted group underwent closed reduction and cannulated screw fixation assisted by TiRobot. The control group received traditional open reduction and plate fixation. No significant differences were found in baseline characteristics between the two groups (*P* > 0.05), ensuring comparability. Data on incision length, operative time, intraoperative blood loss, hospitalization duration, fracture healing time, calcaneal length, width, height, Böhler angle, Gissane angle after complete healing, internal fixation removal, and complications were recorded and compared. At the final follow-up, VAS scores and AOFAS scores with grading were documented. Statistical analysis was performed using IBM SPSS Statistics 26.0. Continuous variables were assessed for normality using the *Shapiro*–*Wilk* test. Normally distributed data were expressed as mean ± standard deviation ($\bar{x}\pm s$) and compared using independent samples t-tests. Non-normally distributed data were presented as median (lower quartile, upper quartile) [*M* (*Q*_1_, *Q*_3_)] and analyzed with Mann–Whitney *U* tests. Categorical data were reported as percentages and compared using Fisher's exact test. A two-sided significance level of *α* = 0.05 was adopted for all statistical tests.

**Results:**

No statistically significant differences were observed between the two groups in terms of calcaneal length, width, height, Gissane angle, AOFAS score grading, postoperative complications, and the number of patients who opted for internal fixation removal after complete fracture healing (*P* > 0.05). The operative time in the robotic group was significantly longer than that in the control group, with a statistically significant difference (*P* < 0.05). The intraoperative blood loss in the robotic group was 17.50 ± 17.68 ml, compared to 45.00 ± 39.79 ml in the control group, indicating that the robotic group had significantly less intraoperative blood loss, with a statistically significant difference (*P* < 0.05). The incision length in the robotic group was 2.30 ± 0.48 cm, while it was 8.10 ± 1.45 cm in the control group, demonstrating that the robotic group had significantly smaller incision lengths, with a statistically significant difference (*P* < 0.05). The hospitalization duration in the robotic group was significantly shorter than that in the control group, with a statistically significant difference (*P* < 0.05). The restoration of Böhler's angle in the robotic group was significantly better than that in the control group, with a statistically significant difference (*P* < 0.05). The fracture healing time in the robotic group was significantly shorter than that in the control group, with a statistically significant difference (*P* < 0.05). The VAS score in the robotic group was 0.40 ± 0.70, compared to 2.00 ± 1.56 in the control group, indicating that the robotic group had significantly lower VAS scores, with a statistically significant difference (*P* < 0.05). The AOFAS score in the robotic group was 94.80 ± 6.21, while it was 85.40 ± 7.99 in the control group, demonstrating that the robotic group had significantly higher AOFAS scores, with a statistically significant difference (*P* < 0.05).

**Conclusion:**

Compared to the control group, TiRobot-assisted minimally invasive treatment for calcaneal fractures significantly reduced intraoperative blood loss and incision length, shortened hospitalization duration, better restored calcaneal anatomy, accelerated fracture healing, more effectively alleviated postoperative pain, and promoted early functional recovery.

## Introduction

The concept of Enhanced Recovery After Surgery (ERAS) has gained increasing acceptance and promotion in surgical fields in recent years. This approach requires clinicians to implement evidence-based perioperative optimization measures to reduce patients' physiological and psychological trauma stress, facilitate rapid recovery from disease and surgical stress, minimize the negative impact of illness and surgery on patients' function and quality of life, reduce complications, and help patients return to normal life better and faster (1).

Calcaneal fractures account for approximately 2% of all fractures, with 60%–75% being displaced intra-articular fractures. Approximately 20% of patients with intra-articular calcaneal fractures fail to return to work within one year (2). Due to the complex anatomy of the calcaneus and surrounding structures, along with poor soft tissue coverage, treatment is challenging and often associated with numerous sequelae and poor prognosis. Regarding surgical approaches, conventional extended lateral L-shaped incisions and sinus tarsi approaches are commonly used for open reduction and internal fixation, though they may lead to complications such as surgical site infection, skin edge necrosis, and sural nerve injury (3, 4). A 20-year follow-up study demonstrated that percutaneous cannulated screw fixation achieved optimal outcomes in foot function, pain relief, and patient satisfaction, suggesting this fixation method is an excellent option for calcaneal fractures with articular displacement, particularly for Sanders type II and III fractures (5). Due to the limitations of human visual perception, manual screw placement is inherently unstable and makes it difficult to ensure optimal positioning and angulation of each screw. Repeated intraoperative fluoroscopic verification and screw repositioning are often required during the procedure. Multiple insertions and withdrawals of screws may compromise local biomechanical stability. The advent of navigation-guided robotic systems has provided a powerful surgical tool for precise screw placement. TiRobot represents one such navigation-guided robotic system.Robot-assisted minimally invasive surgery facilitates early postoperative rehabilitation in patients.

Although studies on TiRobot-assisted minimally invasive treatment of calcaneal fractures have been reported, existing research remains limited with relatively short follow-up periods (6–10). This study conducted a long-term follow-up with an average duration of 27.85 months on 20 patients with calcaneal fractures to further evaluate the efficacy of TiRobot-assisted minimally invasive treatment.

## Methods

### Inclusion and exclusion criteria

Inclusion criteria were as follows:(1) Age ≥18 years; (2) Fractures classified as Sanders type II or III; (3) Follow-up duration ≥12 months; (4) Patients with unilateral calcaneal fractures; (5) The fractures were closed; (6) Patients were willing to undergo either robotic surgery or open reduction and internal fixation and had signed the surgical consent form.

Exclusion criteria were defined as follows: (1) Open calcaneal fractures; (2) Fractures classified as Sanders type I or IV; (3) Unstable vital signs rendering patients ineligible for anesthesia or surgery; (4) Patients with dementia, schizophrenia, or other conditions precluding follow-up compliance; (5) Pathological fractures; (6) There was skin infection at the surgical site; (7) Patients had severe osteoporosis.

A total of 20 patients meeting the selection criteria were enrolled in the study. Based on surgical approaches, they were divided into the robot-assisted group (*n* = 10) and the control group (*n* = 10). No statistically significant differences were observed between the two groups regarding gender, age, body mass index, injury mechanism, injury-to-surgery time, preoperative calcaneal length/width/height, Böhler's angle, Gissane's angle, fracture classification, operative side, or follow-up duration (*P* > 0.05), indicating comparability (Table 1).

**Table 1:** Comparison of baseline data between the two groups.

Baseline data	Robot-assisted group	Control group	Statistical value	*P* value
	(*n* = 10)	(*n* = 10)		
Gender(Male/Female), n	8/2	8/2	—	1.000
Age, years	49.80 ± 17.26	47.10 ± 15.06	*t* = 0.373	0.714
BMI, kg/m^2^	24.73 ± 3.18	22.45 ± 3.38	*t* = 1.551	0.138
Injury mechanism, n			—	1.000
Accidental falls	2	3		
Fall from height	8	7		
Injury-to-surgery time, d	5.50 (3.75, 8.25)	6.00 (3.75, 12.75)	*Z* = −0.380	0.704
Preoperative calcaneal length, mm	73.49 ±5.43	73.22 ± 4.24	*t* = 0.122	0.904
Preoperative calcaneal width, mm	48.99 ± 4.60	48.60 ± 4.72	*t* = 0.189	0.853
Preoperative calcaneal height, mm	41.20 ± 4.43	40.27 ± 3.97	*t* = 0.496	0.626
Preoperative Böhler's angle, °	13.83 ± 5.77	11.76 ± 3.77	*t* = 0.952	0.354
Preoperative Gissane's angle, °	94.75 ± 4.91	95.99 ± 5.67	*t* = −0.521	0.609
Sanders classification (I/II/III/IV), n	0/3/7/0	0/5/5/0	—	0.650
Operative side (left/right), n	3/7	6/4	—	0.370
Follow-up duration, months	30.50 (24.25, 31.25)	30.00 (24.50, 31.25)	*Z* *=* −0.038	0.970

“—”: Fisher's exact test was used, with no statistical value available.

### A brief introduction to TiRobot

**Figure 1:**
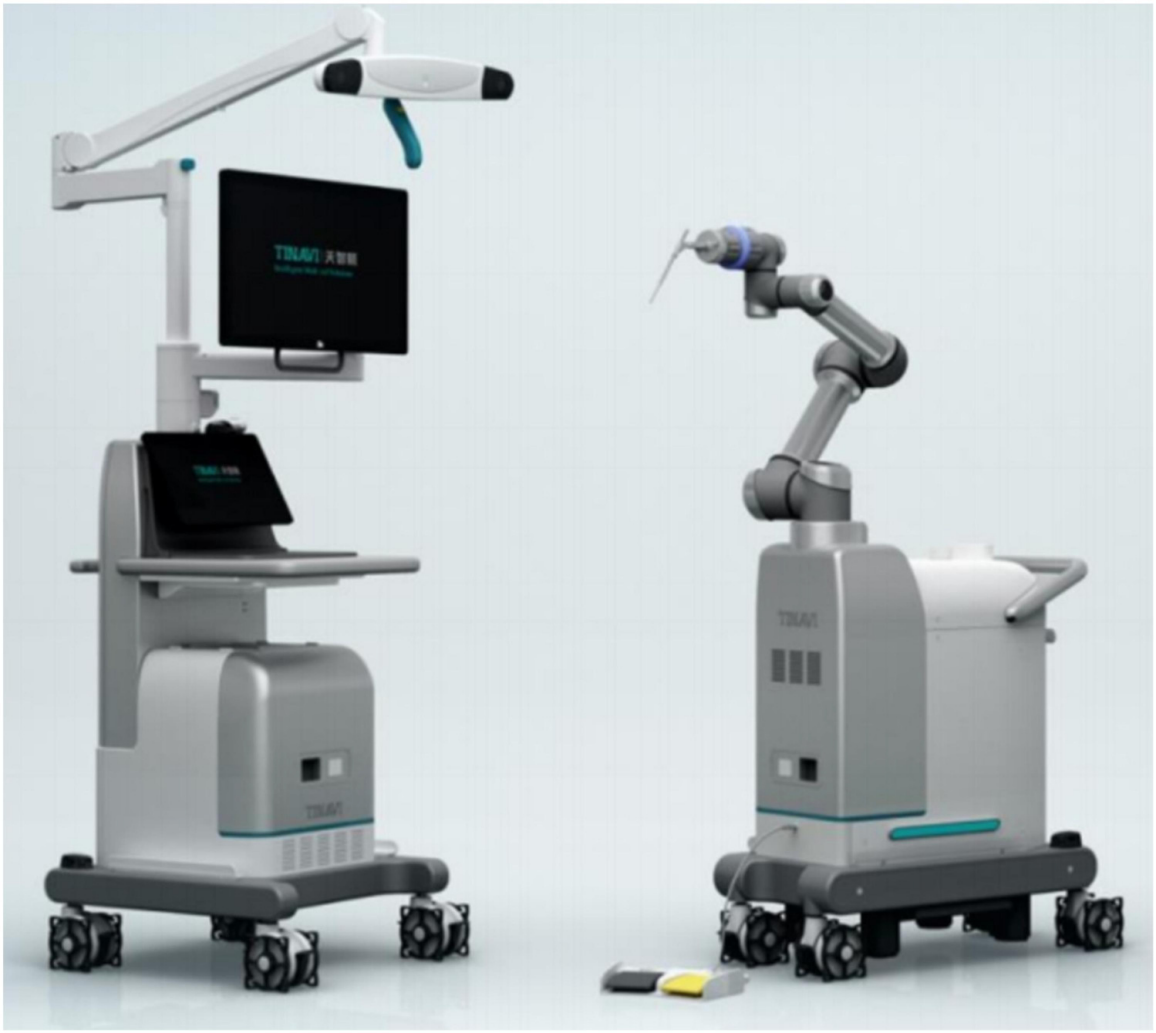
Morphological characteristics of the TiRobot.

The navigation and positioning robot system used in this study was TiRobot, developed by Beijing TINAVI Medical Technologies Co., Ltd., China. TiRobot primarily consisted of a surgical planning platform, optical camera, and a robotic arm (Figure 1).

### Surgical procedure

a. Upon entering the operating room, patients were placed in the prone position. Anesthesia was administered *via* intraspinal block combined with peripheral nerve block. Routine disinfection and draping were performed.
b. The injured foot was securely immobilized using a foot holder, and a tracker was attached to the foot holder.
c. A disposable sterile protective cover was used to establish an aseptic working environment for the C-arm x-ray machine's image intensifier and the robotic arm.
d. Three-dimensional imaging data of the surgical area were acquired using the C-arm x-ray machine and uploaded to the robotic surgical planning platform, where the trajectory for Kirschner wires (K-wires) used in prying reduction was planned.
e. Under robotic guidance, two 2.5 mm K-wires were implanted for prying reduction, and their positions were verified using three-dimensional imaging.
f. A 4.0 mm K-wire was manually inserted for calcaneal traction.
g. With the assistant providing countertraction, the surgeon performed fracture reduction by prying, traction, and manual compression.
h. After reduction, a 2.5 mm K-wire was temporarily inserted to stabilize the fracture, and the reduction was confirmed *via* three-dimensional imaging.
i. Once satisfactory reduction was achieved, another set of three-dimensional images was acquired to plan the length, diameter, and trajectory of cannulated screws based on the patient's actual fracture pattern.
j. After finalizing the surgical path, the orthopedic surgeon stepped on the foot pedal, prompting the robotic arm to automatically move to the planned spatial position. The surgeon then precisely inserted guide pins through the robotic arm's guiding sleeve.
k. Following guide pin placement, three-dimensional imaging verified their positions to ensure alignment with the planned targets. Upon confirmation, cannulated screws were inserted along the guide pins. After all screws were placed, their positions were rechecked *via* three-dimensional imaging. Once all screws were securely positioned as planned, the guide pins were removed.
l. The incision was sutured and dressed with sterile compressive bandaging, concluding the surgical procedure.

### Postoperative management

The postoperative management protocol was identical for both groups. Both groups of patients were immobilized with a cast for two weeks. The affected limb was elevated postoperatively with close monitoring of distal toe circulation. Prophylactic antibiotics were administered for 24 h pre- and postoperatively, along with routine analgesic treatment and regular wound dressing changes. Non-weight-bearing functional exercises of the affected limb were initiated on the operative day. After discharge, patients underwent regular outpatient follow-ups with radiographic examinations. Weight-bearing exercises were progressively increased according to fracture healing status, with full weight-bearing permitted only after confirmed fracture union.

### Efficacy evaluation indices

The following parameters were recorded and compared between the two groups: incision length, operative time, intraoperative blood loss, hospital stay duration, fracture healing time, postoperative calcaneal length/width/height, Böhler's angle and Gissane's angle after complete fracture union, number of patients who underwent implant removal, and complication rates. The criteria for assessing fracture healing are as follows: (1) absence of local tenderness and longitudinal percussion pain; (2) absence of abnormal local movement; and (3) radiographic evidence of continuous callus formation at the fracture site with blurred fracture lines on x-ray images.

At final follow-up, functional outcomes were evaluated using the American Orthopaedic Foot & Ankle Society (AOFAS) Ankle-Hindfoot Scale and categorized as excellent (90–100 points), good (75–89 points), acceptable (50–74 points), or poor (<50 points). Pain levels were assessed in all patients using the VAS score. The AOFAS and VAS scores of all patients were evaluated by independent assessors who were blinded to the group assignments.

### Statistical methods

Statistical analysis was performed using IBM SPSS Statistics 26.0. Continuous variables were assessed for normality using the *Shapiro–Wilk* test. Normally distributed data were expressed as mean ± standard deviation ($\bar{x}\pm s$) and compared using independent samples t-tests. Non-normally distributed data were presented as median (lower quartile, upper quartile) [*M*(*Q*_1_, *Q*_3_)] and analyzed with Mann–Whitney *U* tests. Categorical data were reported as percentages and compared using Fisher's exact test. A two-sided significance level of *α* = 0.05 was adopted for all statistical tests.

## Results

**Figure 2:**
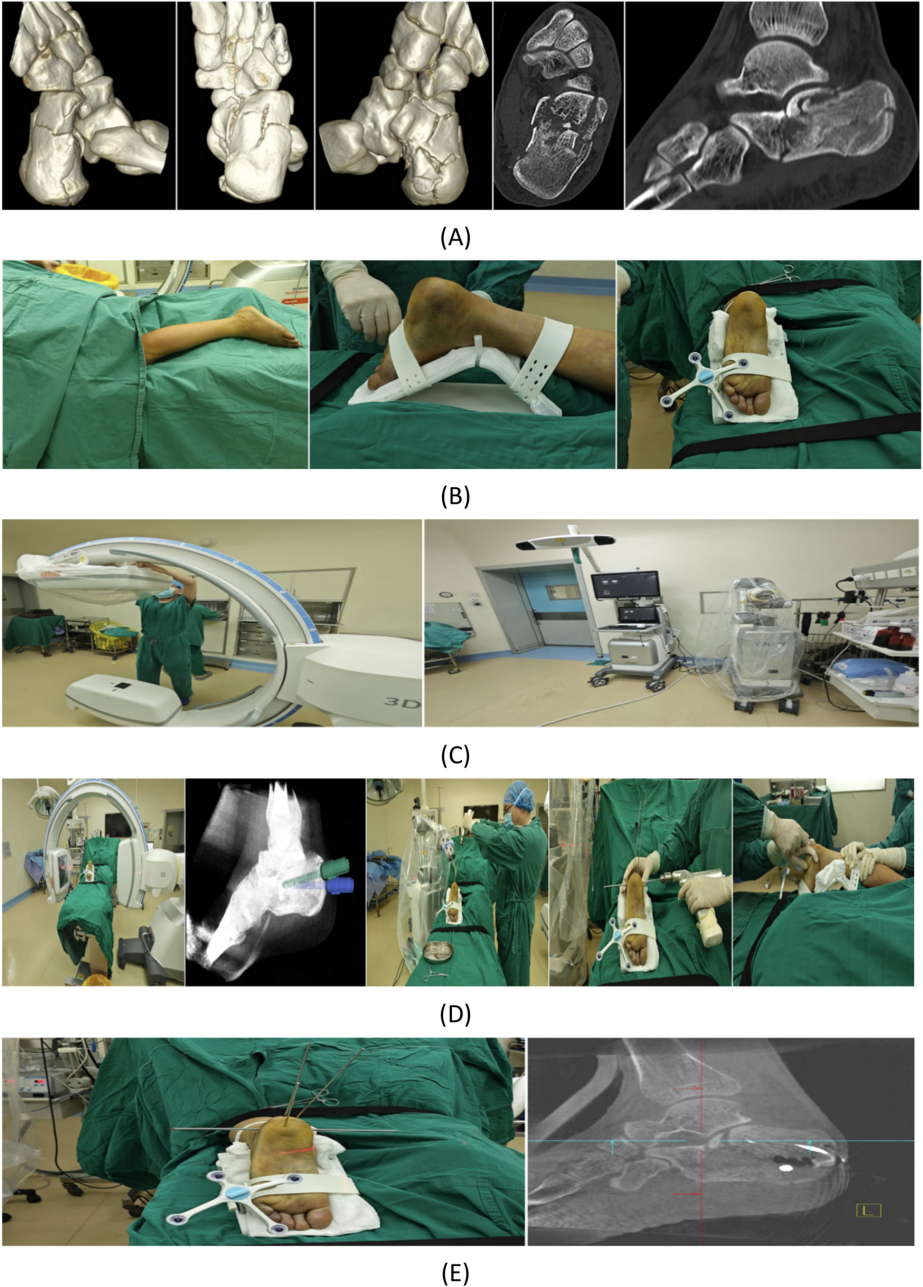

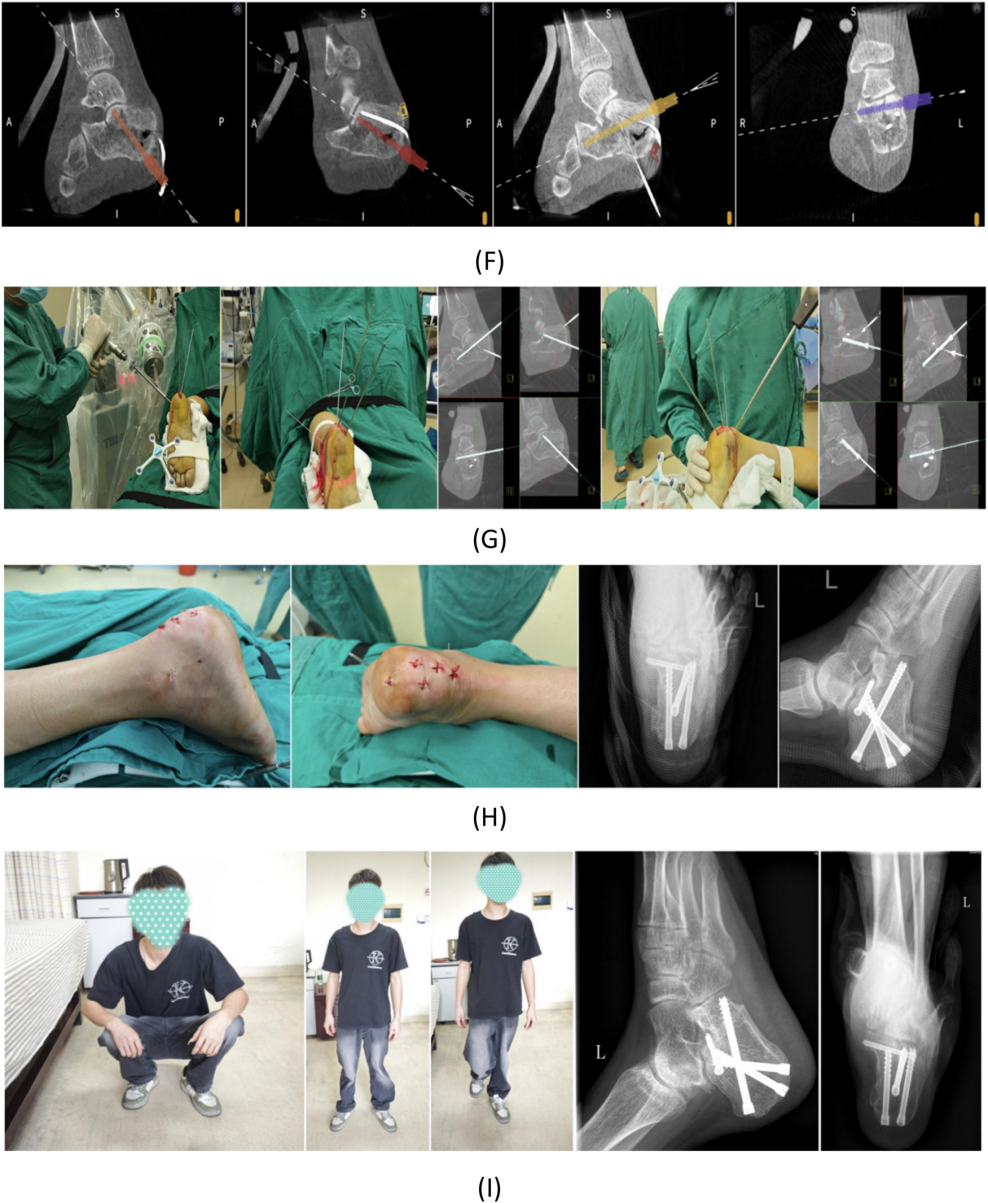
Case presentation: A 24-year-old male patient sustained a left calcaneal fracture due to a fall from height. **(A)** Preoperative CT scan of the calcaneus. **(B)** Routine disinfection and draping were performed. The left foot was securely fixed using a foot holder with a tracker attached. **(C)** Sterile working environments were established for both the C-arm image intensifier and the robotic arm. **(D)** After acquiring 3D intraoperative imaging data and uploading them to the robotic surgical planning platform, trajectories were planned for K-wires used in reduction. Two 2.5 mm K-wires were robotically inserted, followed by manual insertion of a 4.0 mm K-wire for calcaneal traction. After all K-wire placements, closed manual reduction was performed by the surgeon with assistant support. **(E)** Post-reduction, a 2.5 mm K-wire was temporarily placed for fracture stabilization, with reduction quality verified by 3D imaging. **(F)** Four screws were planned for definitive fracture fixation. **(G)** Robot-assisted insertion of four guide pins was performed, with subsequent 3D imaging confirming their positions. Satisfactory guide pin placement was followed by screw insertion and positional verification. **(H)** After confirming proper screw placement, the incision was closed, and postoperative calcaneal radiographs were obtained. **(I)** At 3-month follow-up, complete fracture healing was observed without screw loosening or displacement. The patient demonstrated excellent functional recovery with pain-free ambulation and normal gait.

No statistically significant differences were observed between the two groups in calcaneal length, width, height, Gissane's angle, AOFAS score grading, postoperative complications, or number of patients undergoing implant removal. The robotic-assisted group demonstrated significantly longer operative time, significantly less intraoperative blood loss, significantly smaller incision length, significantly shorter hospital stay, significantly better restoration of Böhler's angle, significantly shorter fracture healing time, significantly lower VAS scores, and significantly higher AOFAS scores compared with the control group. Detailed results are presented in Table 2. Representative case is shown in Figure 2.

**Table 2:** Comparison of outcome measures between the two groups.

Outcome indicators	Robot-assisted group	Control group	Statistical value	*P* value
	(*n* = 10)	(*n* = 10)		
Operation time, min	113.80 ± 37.36	73.50 ± 16.21	*t* = 3.130	0.006
Blood loss, ml	10.00 (5.00, 27.50)	25.00 (10.00, 100.00)	*Z* = −2.099	0.036
Incision length, cm	2.00 (2.00, 3.00)	8.00 (7.00, 8.00)	*Z* = −3.929	0.000
Hospital stay, d	7.00 (6.00, 10.25)	10.00 (8.75, 14.50)	*Z* = −2.138	0.032
Postoperative calcaneal length, mm	76.52 ± 4.97	75.76 ± 3.26	*t* = 0.405	0.691
Postoperative calcaneal width, mm	38.51 (32.76, 39.50)	37.73 (36.20, 38.83)	*Z* = −0.113	0.910
Postoperative calcaneal height, mm	47.24 ± 4.47	48.06 ± 4.17	*t* = −0.424	0.677
Postoperative Böhler's angle	28.83 (25.94, 37.16)	25.19 (21.81, 29.23)	*Z* = −2.117	0.034
Postoperative Gissane's angle	125.48 ± 4.31	117.22 ± 11.67	*t* = 2.099	0.0502
Fracture healing time, months	3.00 (2.00, 4.00)	4.50 (3.78, 5.00)	*Z* = −2.663	0.008
VAS score, points	0.00 (0.00, 1.00)	2.00 (0.00, 3.25)	*Z* = −2.355	0.018
AOFAS score, points	97.50 (89.00, 100.00)	84.50 (79.50, 91.50)	*Z* = −2.436	0.015
AOFAS score Grading, *n*			—	0.070
Excellent	8	3		
Good	2	7		
Acceptable	0	0		
Poor	0	0		
Postoperative complications, *n*(%)				
Surgical site infection	0 (0)	2 (20)	—	0.474
Gait alteration	1 (10)	1 (10)	—	1.000
Traumatic arthritis	0 (0)	1 (10)	—	1.000
Persistent pain	1 (10)	4 (40)	—	0.303
Postoperative removal of internal fixation, n	2	5	—	0.350

“—”: Fisher's exact test was used, with no statistical value available.

## Discussion

Previous studies have demonstrated that the implementation of the Enhanced Recovery After Surgery (ERAS) clinical pathway can significantly reduce patients' length of hospital stay, decrease the incidence of complications and readmission rates, and lower overall healthcare costs (1). Under the ERAS framework, particular emphasis is placed on minimally invasive surgical techniques, soft tissue preservation, precise reduction, and the judicious use of internal fixation devices. In alignment with the ERAS principles, our surgical team successfully performed a minimally invasive closed reduction and internal fixation procedure on the patient, utilizing a series of robot-assisted minimally invasive surgical techniques.

### Brief introduction to TiRobot's key functions

TiRobot features intelligent screw entry point and trajectory algorithms, enabling surgical planning based on either 2D or 3D imaging. It allows simultaneous planning of multiple screw paths with sequential execution, simplifying surgical procedures. The integrated control at the robotic arm's end enables one-click screw selection, improving the efficiency of multi-screw placement. The robotic arm supports motion simulation, allowing surgeons to preview movement trajectories, effectively preventing collisions between the arm and the patient or operating table. After precise positioning by the robotic arm, surgeons can accurately place guid pins and cannulated screws through the guided sleeve at the arm's tip (11, 12). TiRobot features real-time monitoring and self-correction capabilities to maintain consistency between the actual surgical path and the planned trajectory, even if patient positioning shifts during surgery. Its surgical planning interface provides orthopedic surgeons with stereoscopic visualization of screw paths, allowing 3D planning of screw direction, angle, quantity, length, and diameter. The system enables precise supporting fixation of key bone fragments through spatial screw weaving for calcaneal fractures. TiRobot is equipped with a sterile surgical screen for real-time intraoperative planning, an image tracker to enhance imaging efficiency, and an omnidirectional tracker to accommodate multiple surgical positions. The system includes dual foot pedals for seamless switching between manual and automatic modes, with audio-visual prompts at critical surgical steps throughout the procedure.

### Safety analysis of TiRobot in the treatment of calcaneal fractures

The calcaneus resembles an irregular cuboid, and due to physiological hand tremors, it is challenging to manually place screws with high precision. With TiRobot assistance, guide pins and screws can be accurately implanted at a submillimeter level, ensuring screws remain within the bony trajectory, thereby effectively reducing the risk of iatrogenic neurovascular injuries. In this study, all screw placements were successfully achieved in a single attempt. TiRobot incorporates joint force control technology, automatically halting upon encountering obstacles to prevent accidental injuries to both surgeons and patients. Based on TiRobot's workflow, imaging is only required at three stages: preoperatively, after guide pin insertion, and after screw placement, significantly reducing overall x-ray exposure. Cannulated screw fixation minimizes soft tissue disruption, resulting in a low probability of postoperative wound infections and delayed healing. In this study, no cases of wound infection or delayed healing occurred in the robotic group. The tracker is mounted on the foot fixator without causing additional iatrogenic damage. TiRobot is equipped with UPS power backup, ensuring uninterrupted power supply throughout the procedure. If any spatial obstruction occurs between the optical camera and the tracker, TiRobot emits an alert to prevent surgical errors.

### Efficacy analysis of TiRobot-assisted minimally invasive treatment for calcaneal fractures

In terms of operative time, the robotic group exhibited a significantly longer duration compared to the control group. This was primarily attributed to the higher cost of robotic surgery, resulting in fewer patients opting for the procedure and consequently limiting surgeons'experience and proficiency. However, after gaining proficiency, the shortest recorded operative time in the robotic group was 75 min, comparable to that of open reduction and internal fixation with plates.

Regarding intraoperative blood loss, the robotic group demonstrated significantly less bleeding than the control group. The robotic-assisted closed reduction technique minimized soft tissue damage, and TiRobot-guided screw placement within the bony trajectory prevented iatrogenic vascular injuries, further reducing blood loss.

The incision length in the robotic group was significantly shorter than in the control group. Open surgery required extensive soft tissue dissection, causing greater damage to the pericalcaneal tissues. Shorter incisions were crucial in preventing wound infections and delayed healing. In this study, one diabetic patient in each group was included. The diabetic patient in the robotic group showed no postoperative wound infection or delayed healing, whereas the control group's diabetic patient experienced both complications. This was attributed to the robotic-assisted approach, which limited total incision length to 2–3 cm, with each mini-incision averaging only 5 mm. Larger incisions also resulted in more prominent scarring, contributing to postoperative pain and functional limitations in the control group.

Hospital stay was significantly shorter in the robotic group. The minimally invasive closed reduction and internal fixation technique facilitated faster wound healing, reduced observation time, and simplified postoperative wound care.

No significant differences were observed between the two groups in postoperative calcaneal length, width, height, or Gissane's angle after complete fracture healing. However, Böhler's angle restoration was significantly better in the robotic group. TiRobot-assisted Kirschner wire placement in biomechanically optimal positions enabled precise reduction of displaced fragments, restoring the calcaneal anatomy closer to its pre-injury state.

Fracture healing time was significantly shorter in the robotic group. The minimally invasive approach avoided extensive soft tissue dissection, preserving local blood supply and accelerating bone healing. Early healing allowed quicker weight-bearing and functional recovery. One male patient in the robotic group achieved unassisted ambulation by postoperative day 45, demonstrating the advantages of robotic-assisted minimally invasive surgery in rapid rehabilitation.

The robotic group had significantly lower VAS scores than the control group. Cannulated screw fixation, entirely confined within the bone, minimized soft tissue irritation and reduced postoperative pain. In contrast, plate fixation in the control group caused greater soft tissue stimulation due to direct contact, exacerbating discomfort, particularly in the thin and vulnerable lateral calcaneal skin.

AOFAS scores were significantly higher in the robotic group, though both groups achieved a 100% excellent-to-good functional rating without significant differences. This indicated that TiRobot-assisted minimally invasive calcaneal fracture treatment better facilitated functional recovery.

No significant difference was found in postoperative complications between the groups, though the robotic group had a lower absolute incidence. No wound infections or post-traumatic arthritis occurred in the robotic group, likely due to smaller incisions, reduced blood loss, faster healing, and earlier partial weight-bearing, which mitigated arthritis risk.

Notably, control group patients were more inclined to request implant removal after fracture healing, suggesting that plates had a greater impact on daily life and psychological well-being compared to intramedullary cannulated screws.

## Limitations of TiRobot

The main limitations of TiRobot include: (1) lack of fracture reduction capability, only assisting in screw placement after manual reduction; (2) inability to automatically plan screw trajectories, requiring manual planning by surgeons; (3) current inapplicability to Sanders type IV calcaneal fractures with poor mechanical conditions; (4) absence of real-time visualization for guidewire direction and depth during insertion.

## Conclusion

In conclusion, this long-term follow-up study demonstrated that compared with the control group, TiRobot-assisted minimally invasive treatment for calcaneal fractures significantly reduced intraoperative blood loss, shortened incision length and hospitalization duration, better restored calcaneal anatomy, accelerated fracture healing, more effectively alleviated postoperative pain, and facilitated earlier functional recovery. Additionally, the robotic group exhibited less inclination for implant removal than the control group, indicating that cannulated screw fixation imposed lesser impact on patients' daily life and psychological well-being.

Due to the relatively high cost of robotic surgery and the limited number of patients willing to undergo such procedures, the sample size was small, which constrained the statistical power and generalizability of the findings in this study. This study was a single-center retrospective cohort study, which presented limitations such as selection bias and a lack of randomization. Additionally, a certain learning curve was associated with robotic procedures, and due to the limited number of cases, the accumulation of surgical experience was insufficient. In the future, if conditions permit, we aim to conduct a prospective randomized controlled trial in collaboration with multiple medical centers to optimize the study design, increase the sample size, and implement long-term follow-up, thereby enhancing the reliability of the study conclusions.

## Data Availability

The original contributions presented in the study are included in the article/Supplementary Material, further inquiries can be directed to the corresponding author.
